# Severe immune thrombocytopenia following diphtheria, tetanus, pertussis and polio vaccination in a 36-year-old Caucasian woman: a case report

**DOI:** 10.1186/s40001-022-00686-z

**Published:** 2022-05-03

**Authors:** Onno Küster, Jörg Schmohl, Jochen Greiner, Maximilian Andreas Storz

**Affiliations:** 1 MVZ Dillmannstraße, Dillmannstraße 19, 70193 Stuttgart, Baden-Württemberg Germany; 2 Department of Internal Medicine, Diakonie Hospital Stuttgart, 70176 Stuttgart, Germany; 3 Department of Internal Medicine II, Center for Complementary Medicine, Faculty of Medicine, University of Freiburg, 79106 Freiburg, Germany

**Keywords:** Vaccination, Diphtheria, Tetanus, Pertussis, Polio, Immune thrombocytopenia, Bleeding, Platelets, Adverse effect, Case report

## Abstract

**Background:**

Immune thrombocytopenia (ITP) is a rare autoimmune disorder characterized by low platelet counts and increased bleeding risk. The disease may be induced by other disorders, including malignancies, autoimmune diseases, infectious agents or drugs. However, ITP has also been described following vaccinations, such as the measles–mumps–rubella vaccination. In rare cases, ITP may occur in children who received a DTaP-IP (diphtheria, tetanus, acellular pertussis vaccine and inactivated poliovirus) vaccine. Hereinafter, we report the first well-documented cases of ITP in an adult patient in the temporal context of a DTaP-IP vaccination.

**Case presentation:**

This case report attempts to capture the life-threatening picture of a 36-year-old otherwise healthy Caucasian woman with newly diagnosed severe immune thrombocytopenia in the temporal context of a DTaP-IP vaccination. Four days after receiving the vaccine, the women presented to her primary care physician with malaise, fever and recurrent epistaxis. Clinical examination revealed oral petechiae, ecchymoses, and non-palpable petechiae on both legs. The patient was immediately referred to a local hematology unit where she developed hematuria and an intestinal bleeding (WHO Bleeding Grade III) requiring multiple transfusions. After receiving oral corticosteroids and intravenous immunoglobulins, her platelets gradually recovered. Common causes of secondary ITP were ruled out by laboratory investigations, bone marrow and peripheral blood examinations. This raises the possibility of a (secondary) vaccination-associated thrombocytopenia. To the best of our knowledge, this is the first well-documented case of a DTaP-IP vaccination-related ITP in an adult patient in the English literature.

**Conclusion:**

Although a causal connection between both entities may not be established, we would like to raise awareness in clinicians that ITP following DTaP-IP vaccinations is potentially not limited to children, but may also occur in adults. Users of DTaP-IP booster vaccines should be alert of the possibility of such adverse reactions.

**Supplementary Information:**

The online version contains supplementary material available at 10.1186/s40001-022-00686-z.

## Background

Immune thrombocytopenia (ITP) is a rare autoimmune disorder characterized by low platelet counts and an increased bleeding risk [1, 2]. Expertise in the management of affected patients is not widely spread [1] and ITP is usually a diagnosis of exclusion [2]. Patients who develop thrombocytopenia (as defined by a platelet count < 100,000 platelets per microliter) with no clear underlying cause are usually diagnosed with (isolated) primary ITP [2], whereas secondary ITP is defined as an ITP induced by other disorders or treatments [2, 3]. These may include autoimmune disorders [1, 2], solid tumors and lymphoproliferative diseases [4, 5] as well as infectious agents [6], transfusions and drugs (such as interferon) [2, 7]. ITP has also been described in children following vaccinations [8], although this is exceedingly rare [2].

This case reports attempts to capture the clinical picture of a potentially vaccine-associated ITP in an adult patient. In light of the scarce literature with regard to this particular topic, this article intends to elucidate potential barriers to its diagnosis and presents a cases characterized by life-threatening complications due to a vaccine induced ITP.

## Case presentation

A 36-year-old Caucasian woman presented to her primary care physician's office to receive a DTaP-IP booster vaccination (diphtheria and tetanus toxoids and acellular pertussis adsorbed and inactivated poliovirus). Her physical examination and medical history were unremarkable. In the past, she received all recommended vaccinations in accordance with the national immunization schedule developed by the German “Ständige Impfkommission”. The patient was a non-smoker and did not receive any regular medication. Vital parameters were normal and the woman denied any signs of infection. She received the vaccination (“Boostrix Polio”, AC39B145AA, Glaxo Smith Kline, manufactured in Rixensart, Belgium) and was discharged home shortly after.

A few hours later, the woman developed chills, malaise and discomfort. Moreover, she also suffered from agonizing myalgias.

At first, she did not consult a medical professional, but symptoms gradually worsened and 4 days later, she presented again to her doctor’s office after noticing red stains in her mouth and after experiencing epistaxis. The patient denied any signs of blood in urine or stools.

Medical examination revealed multiple oral petechiae (1–2 mm in size) and ecchymoses (approximately 1–2 cm in diameter) as well as multiple flat, non-palpable petechiae on both legs (particularly at the front of both shins). Vital parameters were within the normal range, however, after measuring blood pressure multiple new petechiae appeared on the right arm. Examination of the lungs revealed normal resonance and vesicular breath sounds bilaterally. Heart sounds were normal, without pathological murmurs. Examination of the abdomen revealed the anterior wall to be soft and flat. Bowel sounds were normal and there was no tenderness or palpable mass. Finally, there were no focal neurological deficits.

With signs for severe hemorrhage, the patient was immediately referred to a local hospital with an established hematology unit. Laboratory findings demonstrated severe thrombocytopenia (1000 platelets per microliter of blood; normal range 140,000–440,000/µl) and a normal hemoglobin count (13.4 g/dl; normal range, 11.7–15.7 g/dl). Pseudothrombocytopenia was ruled out using a sodium citrate tube.

Table 1 shows other laboratory hematological parameters that were obtained on admission. The standard biochemistry profile and other laboratory parameters may be obtained from Table 2.

**Table 1:** Laboratory hematological parameters obtained on admission: an overview

Test	Result	Normal range
White blood cells	8.47 × 10^3^/µl	4.0–8.0 × 10^3^/µl
Hemoglobin	13.4 g/dl	11.7–15.7 g/dl
Platelets	1 × 10^3^/µl	140–440 × 10^3^/µl
Hematocrit	39.8%	35–47%
Mean cell volume	93.5 fl	80–96 fl
Mean cell hemoglobin	31.6 pg	26.4–34 pg
Quick	85%	70–100%
International normalized ratio (INR)		0.8–1.2
Activated partial thromboplastin time (aPTT)	41.5 s	26–40 s
Antithrombin 3	88%	70–100%
Fibrinogen	410 mg/dl	200–400 mg/dl
d-Dimer	9.5	0–0.5 µg/ml

**Table 2:** Standard biochemistry profile and other parameters obtained on admission

Test	Result	Normal range
Glucose	124 mg/dl	70–100 mg/dl
Blood urea nitrogen	39 mg/dl	10–50 mg/dl
Creatinine	1.1 mg/dl	0.6–1.3–50 mg/dl
Sodium	138 mmol/l	135–144 mmol/l
Potassium	4.4 mmol/l	3.6–4.8 mmol/l
Chloride	98 mmol/l	94–111 mmol/l
Calcium	2.2 mmol/l	2.0–2.75 mmol/l
Creatine kinase	47 U/l	43–153 U/l
LDH (lactate dehydrogenase)	260 U/l	111–255 U/l
Alkaline phosphatase	37 U/l	42–136 U/l
SGOT	26 U/l	14–34 U/l
SGPT	29 U/l	14–36 U/l
γGT	15 U/l	14–39 U/l
Total serum bilirubin	1.01 mg/dl	0–1,1 mg/dl
Haptoglobin	281 mg/dl	58–356 mg/dl
Lipase	20 U/l	13–60 U/l
C-reactive protein	13.5 mg/dl	0–0.9 mg/dl
Procalcitonin	0.89 µg/l	0–0.5 µg/l
ESR (1 h)	18 mm/h	0–15 mm/h

An electrocardiogram revealed a regular heart rhythm at a rate of 97 beats per minute. Abdominal sonography revealed ascites and a slightly enlarged spleen (12 × 3.5 cm). There were no signs of liver cirrhosis or portal hypertension.

**Fig. 1:**
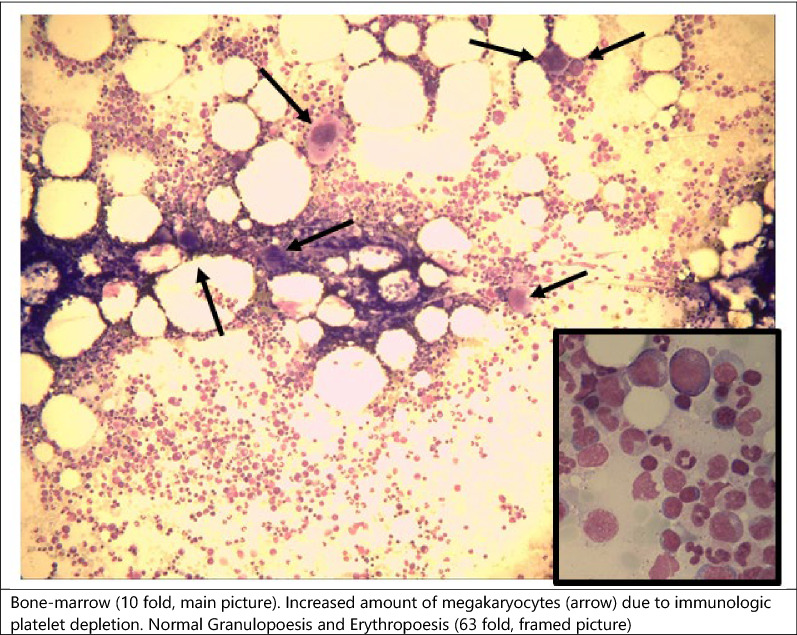
Bone marrow findings

The patient also underwent bone marrow aspiration (Fig. 1). Bone marrow cytology displayed lymphocytes, granulocytes and erythrocytes in normal quantity and morphology. However, megakaryocyte count was significantly elevated with 6–7 /field of view at tenfold magnification (normal range 1–2 megakaryocytes) [9]. Bone marrow immunophenotyping revealed no evidence of lymphoma or significant blast population. Peripheral blood smear showed peripheral depletion of platelets.

Additional blood tests revealed non-elevated antinuclear antibodies (ANA titer < 1:80) and a negative HIV-screening. Furthermore, laboratory tests for Hepatitis B virus, Hepatitis C virus, Epstein–Barr virus, cytomegalovirus, and parvovirus B19 were negative. Screening for HIT (heparin-induced thrombocytopenia) antibodies (targeting the complex of platelet factor 4 and heparin) was negative, as well. Serum vitamin B12 levels and folate were within normal range (Additional file 1).

**Fig. 2:**
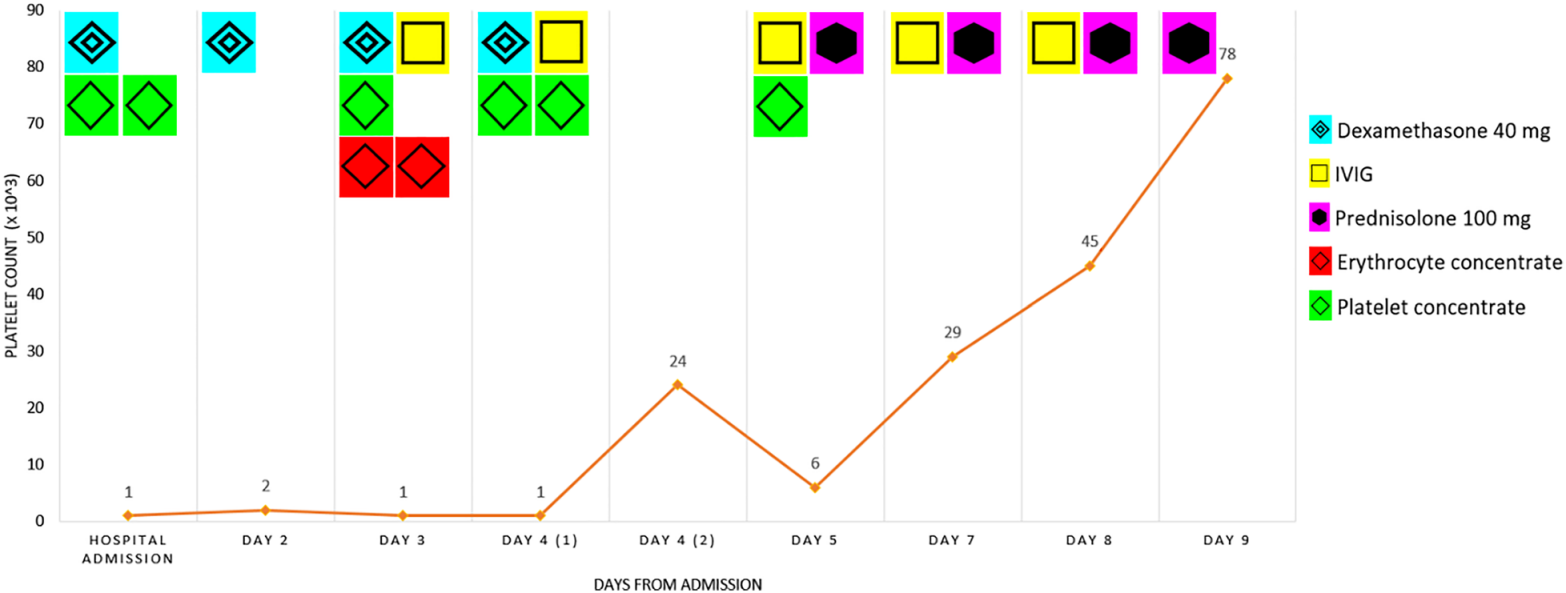
Platelet count timeline

The patient was diagnosed with immune thrombocytopenia and received oral corticosteroids (dexamethasone, 40 mg p.o. daily) in accordance with the 2019 international consensus report on the management of immune thrombocytopenia [10]. As shown in Fig. 2, platelets initially remained low, although dexamethasone (40 mg) was given for 4 days.

Within the next 2 days, the patient developed hematuria. On day 2, she also reported melena and abdominal discomfort. Hemoglobin values dropped from baseline (13.4 g/dl) to 9.1 g/dl and 7.1 g/dl on the third and fourth day, respectively.

Despite a critically low hemoglobin count (5.1 g/dl) in the evening on day 4, the patient refused endoscopy. In light of the substantial clinical bleeding tendency and the low platelet count, the patient was given additional intravenous immunoglobulins (0.5 g/kg per day, see Fig. 2). Furthermore, several platelet concentrates and erythrocyte concentrates were administered.

Under a therapy regimen that included both oral corticosteroids and intravenous immunoglobulins, platelets finally increased on day 5 (Fig. 2). Subsequently, the therapy was changed to prednisolone (p.o., 100 mg daily) on day 5 from admission. Intravenous immunoglobulins were given for 5 days in total. Platelet count increased gradually and the patient was discharged after another 4 days. The patient recovered well without any sequelae.

## Discussion and conclusions

This case report attempts to capture the life-threatening picture of a 36-year-old otherwise healthy woman with newly diagnosed severe immune thrombocytopenia in the temporal context of a DTaP-IP booster vaccination. The patient was hospitalized for a period of 9 days and suffered from recurrent epistaxis, hematuria and an intestinal bleeding (WHO Bleeding Grade III) that required multiple transfusions.

The etiology remains unknown, although many common causes of secondary immune thrombocytopenia were ruled out by laboratory investigations (such as hepatitis, HIV and other viral infections) as well as by bone marrow and peripheral blood examinations (including lymphoproliferative syndromes and immune-mediated thrombocytopenias such as HIT). Moreover, it is also important to emphasize that the patient had an unremarkable past medical history prior to her hospital admission. This raises the possibility of a (secondary) vaccination-associated thrombocytopenia following the DTaP-IP booster vaccination the patient received 3 days prior to hospital referral.

Sudden and severe onset of thrombocytopenia has been occasionally associated with vaccinations [2], however, mainly in children receiving a measles–mumps–rubella vaccine [11]. Furthermore, there are also cases reporting severe thrombocytopenia occurring after influenza vaccinations in adults [12, 13] and after SARS‐CoV‐2 vaccinations with both the Pfizer and Moderna versions [14, 15], although ITP may also be a complication of an infection with COVID-19 itself [16].

Cases of secondary immune thrombocytopenia in the temporal context of DTaP-IP vaccinations are exceedingly rare and, to the best of our knowledge, so far only reported in children and young adolescents [17–20].

Although a causal connection between the vaccination and the ITP may not be established, this case suggests that severe thrombocytopenia can potentially develop secondarily to a DTaP-IP vaccination in adults. Based on an extensive literature research using two scientific databases (PubMed and Google Scholar), we conclude that this is one of the first well-documented cases of immune thrombocytopenia in an adult patient in the temporal context of DTaP-IP vaccination.

ITP is an autoimmune disorder characterized by autoantibodies that inhibit platelet production and impair circulating platelets [21, 22]. This leads to thrombocytopenia and subsequent bleeding. ITP may be found secondary to other conditions, such as infections, but it has also been rarely described following different types of vaccinations [23]. David and Shoenfeld recently summarized potential pathways how vaccines could lead to ITP [23]. These include molecular mimicry, epitope spreading, and finally polyclonal activation, especially in cases of attenuated virus stimulation [23–25]. Apparently, genetic predisposition may also potentially play a role, as well [26].

While it is beyond the scope of this case report to discuss molecular pathways in detail, we believe that this case is of paramount importance for clinicians, as it emphasizes the possibility of a potentially vaccine-associated ITP in the context of DTaP-IP vaccination in an adult.

Although a recent Japanese study suggested no significant ITP risk following vaccinations or simultaneous vaccination in any age group [27], we would like to raise awareness that ITP following DTaP-IP vaccinations may not be limited to children but can potentially occur in adults as well. Prescribers and users of DTaP-IP booster vaccines should be alert of the possibility of such adverse reactions.

## Supplementary Information

**Additional file 1.** The patient kindly declined to share her perspective on the treatment(s) she received. The CARE guidelines were followed for this case report. The CARE Guidelines Checklist is available as a supplementary file. The ISPE guidelines for submitting adverse event reports for publication were followed, as well.

## Data Availability

All data associated with this paper will be made available upon reasonable request.

## Abbreviations

DTaP-IP: Diphtheria and tetanus toxoids and acellular pertussis adsorbed and inactivated poliovirus
ITP: Immune thrombocytopenia
