# Supplementary material for: Severe immune thrombocytopenia following diphtheria, tetanus, pertussis and polio vaccination in a 36-year-old Caucasian woman: a case report
Source: Eur J Med Res. 2022 May 3;27:63. doi: 10.1186/s40001-022-00686-z (PMC9062629; doi:10.1186/s40001-022-00686-z)
Supplement: Supplementary file 1 — Additional file 1. The patient kindly declined to share her perspective on the treatment(s) she received. The CARE guidelines were followed for this case report. The CARE Guidelines Checklist is available as a supplementary file. The ISPE guidelines for submitting adverse event reports for publication were followed, as well. [file 40001_2022_686_MOESM1_ESM.pdf]

CARE Checklist of information to include when writing a case report

CARE case report guidelines

(cc) BY-NC-ND

Topic	Item	Checklist item description	Reported on Line
Title	1	The diagnosis or intervention of primary focus followed by the words "case report"	Page 1, Line 4
Key Words	2	2 to 5 key words that identify diagnoses or interventions in this case report, including "case report"	Page 3, Lines 51ff
Abstract (no references)	3a	Introduction: What is unique about this case and what does it add to the scientific literature?	Page 2, Line 26
	3b	Main symptoms and/or important clinical findings	Page 2, Lines 33ff
	3c	The main diagnoses, therapeutic interventions, and outcomes	Page 2, Line 38
	3d	Conclusion—What is the main "take-away" lesson(s) from this case?	Page 3, Line 47
Introduction	4	One or two paragraphs summarizing why this case is unique (**may include references**)	Page 4, Line 68
Patient Information	5a	De-identified patient specific information.	Page 4, Line 73
	5b	Primary concerns and symptoms of the patient.	Page 4, Line 82
	5c	Medical, family, and psycho-social history including relevant genetic information	Page 4, Line 75
	5d	Relevant past interventions with outcomes	Page 4, Line 77
Clinical Findings	6	Describe significant physical examination (PE) and important clinical findings.	Page 5, Line 88
Timeline	7	Historical and current information from this episode of care organized as a timeline	Page 9, Line 137
Diagnostic Assessment	8a	Diagnostic testing (such as PE, laboratory testing, imaging, surveys).	Page 5, Line96 ff
	8b	Diagnostic challenges (such as access to testing, financial, or cultural)	n/a
	8c	Diagnosis (including other diagnoses considered)	Page 9, Line 133
	8d	Prognosis (such as staging in oncology) where applicable	n/a
Therapeutic Intervention	9a	Types of therapeutic intervention (such as pharmacologic, surgical, preventive, self-care)	Page 9, Line 131 ff
	9b	Administration of therapeutic intervention (such as dosage, strength, duration)	Figure 2
	9c	Changes in therapeutic intervention (with rationale)	n/a
Follow-up and Outcomes	10a	Clinician and patient-assessed outcomes (if available)	n/a
	10b	Important follow-up diagnostic and other test results	Page 9, Line 125ff
	10c	Intervention adherence and tolerability (How was this assessed?)	n/a
	10d	Adverse and unanticipated events	n/a
Discussion	11a	A scientific discussion of the strengths AND limitations associated with this case report	Page 10, Line 161ff
	11b	Discussion of the relevant medical literature **with references**.	Page 10, Line 169ff
	11c	The scientific rationale for any conclusions (including assessment of possible causes)	Page 10, Line 184ff
	11d	The primary "take-away" lessons of this case report (without references) in a one paragraph conclusion	Page 11, Line 18
Patient Perspective	12	The patient should share their perspective in one to two paragraphs on the treatment(s) they received	n/a (patient declined)
Informed Consent	13	Did the patient give informed consent? Please provide if requested	Yes ☒ No ☐
